# Ultrasound-Guided Interscalene Catheter Complicated by Persistent Phrenic Nerve Palsy

**DOI:** 10.1155/2018/9873621

**Published:** 2018-01-03

**Authors:** Andrew T. Koogler, Michael Kushelev

**Affiliations:** Department of Anesthesiology, The Ohio State University Wexner Medical Center, 410 W. 10th Ave., Columbus, OH 43210, USA

## Abstract

A 76-year-old male presented for reverse total shoulder arthroplasty (TSA) in the beach chair position. A preoperative interscalene nerve catheter was placed under direct ultrasound-guidance utilizing a posterior in-plane approach. On POD 2, the catheter was removed. Three weeks postoperatively, the patient reported worsening dyspnea with a subsequent chest X-ray demonstrating an elevated right hemidiaphragm. Pulmonary function testing revealed worsening deficit from presurgical values consistent with phrenic nerve palsy. The patient decided to continue conservative management and declined further invasive testing or treatment. He was followed for one year postoperatively with moderate improvement of his exertional dyspnea over that period of time. The close proximity of the phrenic nerve to the brachial plexus in combination with its frequent anatomical variation can lead to unintentional mechanical trauma, intraneural injection, or chemical injury during performance of ISB. The only previously identified risk factor for PPNP is cervical degenerative disc disease. Although PPNP has been reported following TSA in the beach chair position without the presence of a nerve block, it is typically presumed as a complication of the interscalene block. Previously published case reports and case series of PPNP complicating ISBs all describe nerve blocks performed with either paresthesia technique or localization with nerve stimulation. We report a case of a patient experiencing PPNP following an ultrasound-guided placement of an interscalene nerve catheter.

## 1. Introduction

Interscalene blocks (ISB) are frequently used as an adjuvant therapy for shoulder surgery to optimize postoperative pain, decrease the length of hospitalization, and minimize the time in the postanesthesia care unit [1]. Up to 100% of patients receiving an ISB can anticipate transient phrenic nerve palsy with full recovery following nerve block resolution [2].

Prolonged phrenic nerve paresis (PPNP) resulting from interscalene catheters is rare. One previous investigation identified cervical degenerative disc disease as a potential risk factor for developing PPNP following ISB [3]. Otherwise, there have been few other proposed risk factors thought to contribute to the development of PPNP. We present a case of PPNP following placement of an interscalene nerve catheter for a patient undergoing reverse total shoulder arthroplasty (TSA) with previously undiagnosed cervical degenerative disc disease. This case report is to help highlight risk factors for the development of PPNP, as well as the appropriate work-up and treatment of PPNP.

## 2. Case Report

Verbal and written permission was obtained from the patient for the publication of this report. Written permission was obtained for the use of the patient's medical records.

A 76-year-old male of average body habitus with history of hypertension, chronic obstructive pulmonary disease (COPD), coronary artery disease, and myocardial infarction status after three percutaneous coronary interventions presented for reverse TSA in the beach chair position under general anesthesia with an interscalene nerve catheter for postoperative pain management. After obtaining surgical and anesthetic consent, the patient was placed in a semirecumbent position with his head turned slightly toward the nonoperative shoulder with care to avoid neck discomfort. Intravenous midazolam and fentanyl were administered to achieve moderate sedation for the placement of an interscalene nerve catheter under direct ultrasound-guidance utilizing a lateral-to-medial in-plane approach as described by Antonakakis et al. [4]. A 17-gauge 5 cm Tuohy needle (Arrow International, Reading, PA, USA) was inserted at the anterior edge of the trapezius muscle and advanced using an in-plane technique, passing through the middle scalene muscle and entering the interscalene groove. An initial bolus of 30 cc of 0.5% ropivacaine was delivered in the interscalene groove on the posterior edge of the C5 and C6 nerve roots, followed by the advancement of a 19-gauge Arrow Stimucath Catheter (Teleflex Medical, Reading, PA, USA) without resistance 3 cm past the needle tip. The Tuohy needle was subsequently removed. A 5 ml test dose of 1.5% lidocaine with 1 : 200,000 epinephrine was administered followed by injection of 10 cc of 0.5% ropivacaine with direct ultrasound visualization of the injectate along the posterior edge of the interscalene groove. The catheter was secured with Dermabond liquid adhesive (Ethicon, Somerville, NJ, USA) and covered with a Tegaderm dressing (3M Company, St. Paul, MN, USA). The patient was responsive to verbal stimulation and denied paresthesia or shortness of breath immediately after catheter placement. He was then brought to the operating room and connected to standard anesthesia monitors. General anesthesia was induced with fentanyl, propofol, and rocuronium followed by an uneventful placement of a 7.0 endotracheal tube with direct laryngoscopy. Intraoperatively the patient was placed in the beach chair position with his neck in a neutral position with the combination of a head rest and towels below the jaw line after the endotracheal tube was secured. Neutral head positioning was reconfirmed throughout the course of the surgery by the anesthesia provider. Total surgery time was exactly three hours. Patient was extubated and transferred to the recovery room. One hour postoperatively in the postanesthesia recovery unit, the interscalene catheter was attached to an Elastomeric ON-Q pump (I-Flow Corporation, VQ OrthoCare, Irvine, CA, USA) followed by a 2-day infusion of 0.2% ropivacaine at 10 ml/hour. The patient's surgical course was uneventful, and he was seen on postoperative days (POD) 1 and 2 during acute pain rounds. The patient did not endorse any significant changes in his baseline exertional dyspnea, and his pain was well controlled. On POD 2, the catheter was removed, and he was discharged without significant complaints.

Three weeks postoperatively, the patient presented to his PCP with a complaint of worsening dyspnea. He was diagnosed with bronchitis and prescribed a course of antibiotics and inhalers. After completing his antibiotic regimen, the patient continued to experience dyspnea and was referred to a pulmonologist for consultation. Further investigation revealed that the patient had both orthopnea and worsened dyspnea on exertion. The physical exam revealed decreased breath sounds in the right lower lobe lung field and bilateral upper airway expiratory wheezing. A chest X-ray (CXR) demonstrated an elevated right hemidiaphragm with a small right-sided pleural effusion.

**Figure 1:**
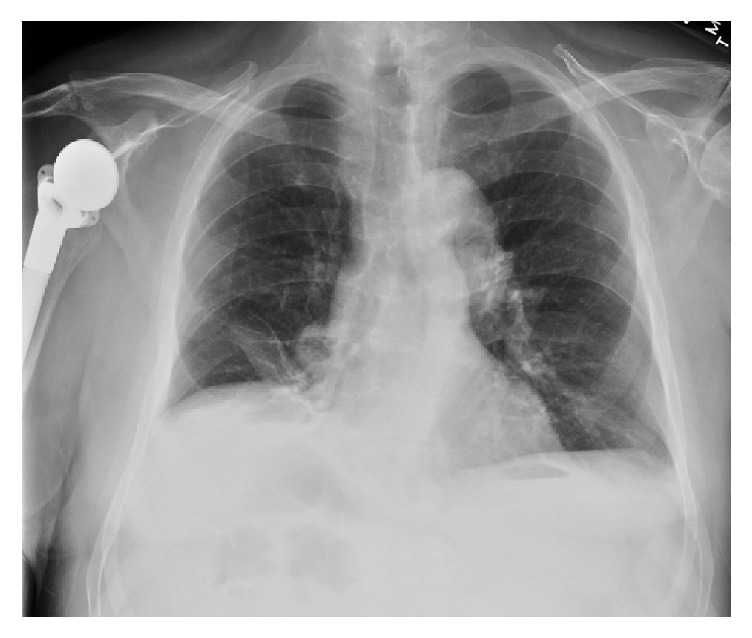
Chest X-ray 6 weeks postoperatively.

**Figure 2:**
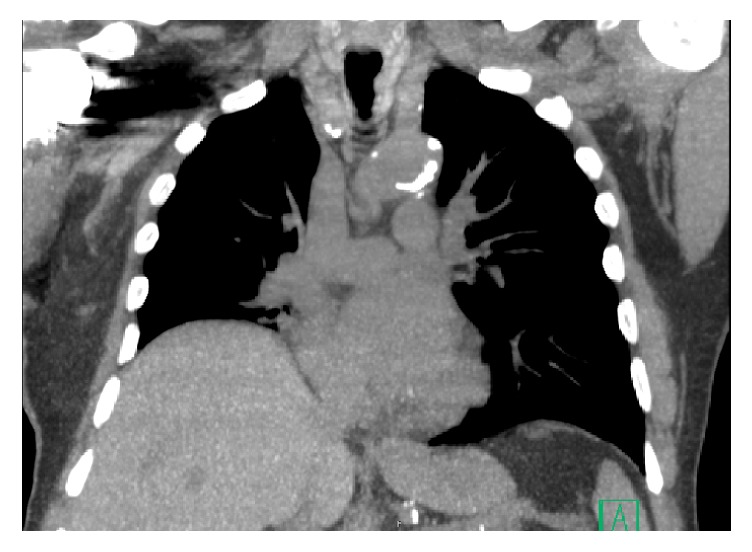
Chest CT scan 6 weeks postoperatively.

**Figure 3:**
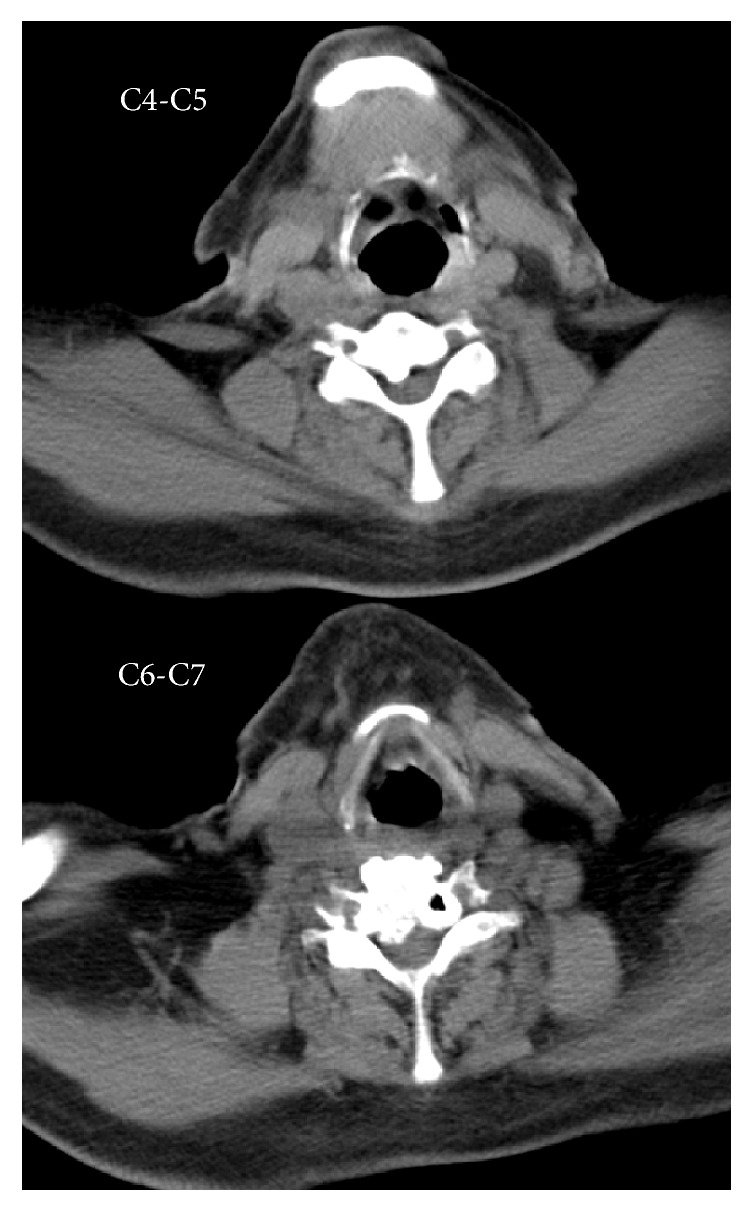
Neck CT scan C4-C5, C6-C7.

**Figure 4:**
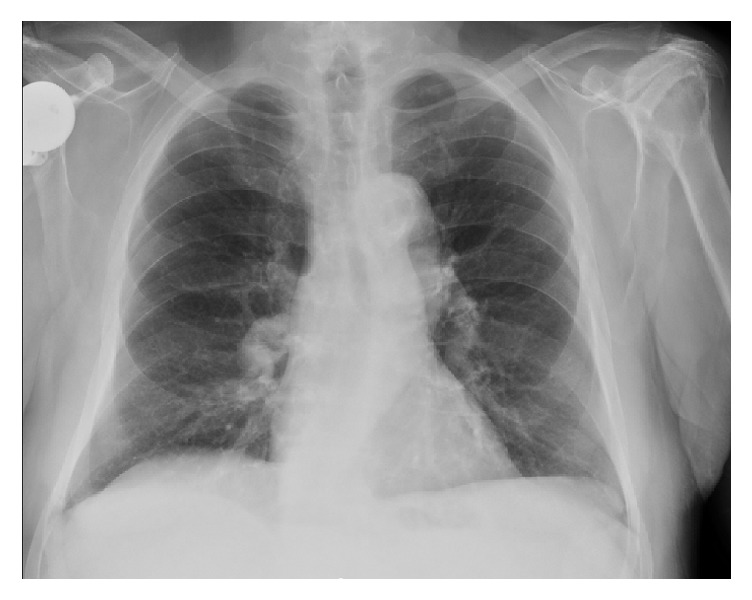
Chest X-ray 1 year postoperatively.

A repeat CXR six weeks postoperatively continued to demonstrate an elevated right hemidiaphragm and right-sided pleural effusion, unchanged from his previous CXR (Figure 1). A diaphragm fluoroscopy (sniff test) confirmed a paralyzed right hemidiaphragm. Chest (Figure 2) and neck CT scans demonstrated an elevated right hemidiaphragm with overlying right lower lobe atelectasis and degenerative changes in his cervical spine (C4–7) consistent with cervical spinal stenosis (Figure 3). The patient had repeat pulmonary function testing, which demonstrated a worsening deficit from presurgical values (Table 1). The patient decided to continue conservative management consisting only of close follow-up with his pulmonologist and declined further invasive testing or treatment, such as an EMG or surgical intervention. The patient was followed for one year postoperatively with moderate improvement of his exertional dyspnea. A repeat CXR at 1 year postoperatively no longer demonstrated an elevated right hemidiaphragm, although the patient continued to endorse mildly worsened exertional dyspnea compared to preoperative levels (Figure 4).

## 3. Discussion

The anatomical proximity of the brachial plexus and phrenic nerve leads to a nearly universal transient blockade of the phrenic nerve with large volume ISB; however, PPNP is a rare complication with a reported incidence to be 1 out of every 2069 single shot ISB or 0.048% [3]. The close proximity of the phrenic nerve to the brachial plexus in combination with its frequent anatomical variation can lead to unintentional mechanical trauma, intraneural injection, or chemical injury during performance of ISB [5]. Our patient's phrenic nerve was not readily identifiable on a brief preprocedure ultrasound (US) examination. Various in-plane and out-of-plane techniques have been described for performance of the continuous interscalene block without any clear consensus as to the technique that is most efficacious [6]. The needle trajectory for the in-plane technique can be either lateral-to-medial or medial-to-lateral. Neither approach eliminates risk of nerve injury as the dorsal scapular nerve (DSN) and the long thoracic nerve (LTN) travel through the middle scalene muscle, while the phrenic nerve may be placed at risk of injury while traversing along the anterior scalene muscle. Out-of-plane approaches place the needle trajectory in closer proximity to the phrenic nerve, potentially increasing the risk of mechanical trauma. US guidance may allow for visualization of the DSN, LTN, and phrenic nerve, therefore limiting the risk of nerve injury [7].

Although decreasing the volume of local anesthetic utilized for interscalene blockade has been shown to decrease the incidence transient phrenic nerve palsy [8], total dose or volume of local anesthetic has not been identified as a risk factor for developing PPNP [3]. Additionally, relatively large initial block volumes of 40 to 65 mL are routinely used to establish interscalene blockade in the era of US guidance [9]. With the use of relatively large initial volumes, our patient must have certainly experienced phrenic nerve palsy during the course of his interscalene catheter infusion (approximately 50 hours); however, his baseline preoperative dyspnea, relative inactivity as an inpatient, and accessory muscle utilization during his 2-day postoperative hospitalization are thought to have masked a worsening of his exertional dyspnea. Although partially compensated phrenic nerve palsy was to be anticipated, there were no indications to suggest development of PPNP prior to discharge from the hospital.

Proposed mechanisms for PPNP complicating an ISB include compression neuropathy from needle trauma, intraneural injection, chemical toxicity, or neuronal ischemia [10, 11]. Shoulder arthroplasty as well as beach chair positioning, separate from regional anesthesia, has been associated with nerve injury at a rate of 0.6–3.6%, most commonly involving the axillary or musculocutaneous nerves. Mechanism of injury is often related to direct trauma, retraction, hematoma formation, or neck positioning during surgical manipulation [12]. Incidentally, TSA in the beach chair position without a regional nerve block has been reported to result in a PPNP in a case that did not involve a regional anesthetic [13]. Ultimately the definitive cause of PPNP cannot be determined without electromyography or a pathological analysis of the phrenic nerve. One hypothesis describing such an unexpected persistent nerve injury is the “double-crush” phenomenon [14]. Our patient may have suffered a neural insult secondary to the regional anesthetic technique, combined with compression of the phrenic nerve at the root level secondary to surgical positioning, traction, or underlying (at the time unknown) C4–C7 cervical degenerative disease.

Previously published case reports and case series of PPNP complicating ISBs all describe nerve blocks performed with either paresthesia technique or localization with nerve stimulation (NS). Of note, this patient's interscalene catheter was performed with only US guidance and did not rely on NS or paresthesia techniques. Although the use of US guidance for regional anesthesia has not demonstrated a reduction in peripheral nerve injury, routine use of US guidance allows practitioners many practical advantages [15]. A recent review demonstrated a decreased number of needle passes, decreased procedural time, and decreased procedure pain for US guided blocks as compared to NS guided blocks [16]. Furthermore, the ability to observe “real-time” needle advancement suggests the potential for US guidance to decrease nerve injury, including the phrenic nerve, compared to other localization techniques. In support of such a hypothesis, a letter to the editor in response to the case-control series identifying risk factors for PPNP speculated that persistent phrenic nerve dysfunction may disappear as a complication of ISB with the transition to US guided needle localization [17]. Unfortunately, we report that exclusive US guidance for performance of interscalene catheter placement did not eliminate the rare complication of PPNP following ISB. Utilizing NS in addition to US guidance would have potentially elicited a diaphragmatic muscular contraction during catheter placement. However, NS has demonstrated low sensitivity for detecting direct needle-to-nerve contact; therefore, NS may not have made a significant difference in this case in preventing PPNP [18]. In addition, relying partially on NS may lead to a greater number of needle passes making the patient more susceptible to potential phrenic nerve trauma [16].

We attempt to add to the body of literature describing the phenomenon of PPNP following ISB. The only previously identified risk factor for PPNP is cervical degenerative disc disease [3]. Our patient was diagnosed with degenerative disc disease following development of postoperative PPNP. Various diagnostic studies including CXR, diaphragm fluoroscopy, spirometry, nerve conduction testing, and electromyography can be used to diagnose phrenic nerve palsy, as well as assessing respiratory improvement during recovery. The long course of the phrenic nerve and slow rate of nerve regeneration may allow for improvement of PPNP up to 24 months after initial injury [19]. Surgical decompression with or without nerve grafting has shown to improve 69% of PPNP cases that did not improve with conservative treatment [20]. The time course for nerve regeneration coincides with this patient's slow resolution of dyspnea over 12 months and supports the case of phrenic nerve disruption with eventual regeneration. Anesthesiologists should be aware of the risk factors that may place patients at a higher likelihood of developing PPNP. Cautious patient selection and close postoperative monitoring should be considered given the significant consequences patients may face from persistent phrenic nerve palsy.

## Figures and Tables

**Table 1:** Pulmonary function tests pre- and postoperatively.

	Preoperative			Postoperative		
	Pred	Actual	% Pred	Pred	Actual	% Pred
Forced vital capacity (FVC; L)	3.86	3.39	88	3.59	2.12	59
Forced expiratory volume 1 (FEV1; L)	2.81	2.18	78	2.56	1.29	50
% FEV1/FVC	73.0%	64.3%		72.0%	61.0%	
Mid-expiratory flow (FEF25–75; L/sec)	2.10	0.99	47	1.71	0.58	32
Peak flow (PF; L/sec)	7.49	6.46	86	6.84	6.01	88
